# Novel Non-coding RNA Analysis in Multiple Myeloma Identified Through High-Throughput Sequencing

**DOI:** 10.3389/fgene.2021.625019

**Published:** 2021-05-24

**Authors:** Minqiu Lu, Yin Wu, Wen Gao, Ying Tian, Guorong Wang, Aijun Liu, Wenming Chen

**Affiliations:** ^1^Department of Hematology, Beijing Chao-Yang Hospital, Capital Medical University, Beijing, China; ^2^Department of Hematology, Beijing Jishuitan Hospital, Fourth Medical College of Peking University, Beijing, China

**Keywords:** non-coding RNAs, multiple myeloma, sequencing, oncogene, ceRNA

## Abstract

This study aimed to explore the potential effects of novel non-coding ribose nucleic acids (ncRNAs) in patients with multiple myeloma (MM). The gene expression profile of plasma cells was used for sequence analysis to explore the expression pattern of ncRNAs in MM. The expression patterns of non-coding RNAs in MM were analyzed by RNA sequencing (whole-transcriptome-specific RNA sequencing). Next, the expression of the selected ncRNAs was verified by quantitative real-time polymerase chain reaction. Further, the lncRNA-associated competitive endogenous RNA network in MM was elucidated using deep RNA-seq. Differentially expressed (DE) ncRNAs were significantly regulated in patients with MM. DE target lncRNAs were analyzed by *cis* and *trans* targeting prediction. Two new lncRNAs were shown to be related to MM oncogenes. MSTRG.155519 played a carcinogenic role in myeloma by targeting CEACAM1; MSTRG.13132 was related to FAM46C. Finally, the network of lncRNA–mRNA–miRNA in MM was constructed in this study. The expression of non-coding RNAs through sequence and functional analyses might be helpful for further studies on the pathogenesis of MM and the development of new MM-targeted therapy for non-coding RNAs.

## Introduction

Multiple myeloma (MM) is a plasma cell neoplasm characterized by the clonal proliferation of malignant plasma cells in the bone marrow microenvironment, clonal proliferation of monoclonal proteins in the blood or urine, and organ dysfunction. The established high-dose therapy/autologous stem cell transplantation and novel drug therapy for MM improved the rate of complete response significantly. However, most patients experience a relapse and require additional treatment. Therefore, it is particularly urgent to explore the new mechanism of MM and develop effective drugs. In addition, the current evaluation of the therapeutic effect on MM is MRD (minimal residual disease) negative. Even if MRD is detected negative by the existing technology, whether the disease is eradicated cannot be determined. Therefore, in addition to the existing detection indicators, other detection items should also be explored to evaluate the MRD-negative result.

Studies of genomics and epigenomics demonstrated that abnormal expression and dysfunction of non-coding ribose nucleic acids (ncRNAs) were important in the pathogenesis of human cancers, similar to protein-coding genes. The human genome shows that non-coding proteins account for 98% of the genome (Fishilevich et al., 2017). ncRNAs can be divided into long non-coding RNAs (lncRNAs; with a length >200 bp) and microRNAs (miRNAs; <200 nucleotides long) according to their sizes (Friedman et al., 2009; Winter et al., 2009; Cech and Steitz, 2014; Fatica and Bozzoni, 2014; Johnsson et al., 2014; Forrest and Khalil, 2017). These ncRNAs have been presumed to be the important new regulators of genetic expression under different pathological conditions (Arjumand et al., 2018; Drak Alsibai and Meseure, 2018). The main mechanisms and functions of lncRNAs include the regulation of gene transcription and translation, epigenetic modification, and interaction with RNA-binding proteins (Tsai et al., 2010; Guttman et al., 2011; Wang and Chang, 2011; Hu et al., 2018). In addition, the regulation of miRNA is also a major mechanism of lncRNAs. lncRNAs regulate the function of miRNA through a competitive endogenous RNA (ceRNA) network that works as an miRNA sponge (Karreth et al., 2015; Zhang and Huang, 2015). Recent studies showed that circRNA served as a new ncRNA, which had been proven to contain target sites for miRNAs and played a role of miRNA sponge in various diseases by disturbing the miRNA signal axis (Zhang et al., 2018).

In recent years, the research on lncRNAs in MM has been emphasized. The knowledge of the key regulators of MM behavior facilitates the development of more effective therapeutic approaches. In this regard, lncRNAs may be involved in regulating the biological function of MM by increasing evidence. One study showed an aberrant expression of multiple lncRNAs in more aggressive stages of MM, suggesting that lncRNAs were vital in the progression of MM (Ronchetti et al., 2016). Another study by Ronchetti et al. (2018) provided a special view for the expression of lncRNA in MM. They examined 30 patients with MM and found a significant correlation between the natural clustering of the transcriptional configuration of whole lncRNAs and the chief molecular prognostic change in MM. In a previous study, an abnormal expression of known lncRNAs in patients with MM was found, including maternally expressed gene 3 (MEG3), colon cancer–associated transcript 1 (CCAT1), and coiled-coil domain-containing 26 (CCDC26) (Lu et al., 2019). Recently, the expression of circRNA in MM was detected using microarray, but the research on the ceRNA of circRNA was lacking (Zhou et al., 2020).

However, the study of the mode of action of lncRNAs in MM is still limited, the expression pattern of ncRNAs in MM is still unclear, and targeted therapies are unavailable. In this study, the sample size increases, and the ncRNAs of MM were predicted and analyzed by RNA second-generation sequencing technology. At the same time, deep RNA-seq was used to elucidate the ceRNA network of MM. This study found more differentially expressed (DE) genes in MM, especially unpublished lncRNAs, using second-generation sequencing, and lncRNA of the MM database was expanded. In particular, new RNAs related to MM pathogenic genes were identified and verified, providing the experimental basis for new targeted therapies for MM.

## Materials and Methods

### Patients and Cell Samples of Bone Marrow

The plasma cells of bone marrow and normal cells were collected from 10 patients with newly diagnosed MM. The patients in the present study met the updated diagnostic criteria of the International Myeloma Working Group for MM (Rajkumar et al., 2014). The median age of the patients was 63.5 (53–72 years). The bone marrow puncture was performed in 10 patients (six males and four females) during the initial diagnosis. Moreover, the CD138 magnetic beads were extracted from myeloma cells, and the transcriptome sequencing was performed. MNCs (mononuclear cells) were extracted from 5 mL of bone marrow. Then, 10 μL of anti-CD138 antibody and 40 μL of PBS were added to every 10^7^ MNCs and then incubated for 15–20 min at 4°C after blending. The MS column was immobilized in the magnetic bead cell sorting field. The cell suspension was added into the MS column after being washed with PBS buffer and separated. After negative cell collection, 0.5 mL of PBS buffer was added to wash the column two times. The MS column was removed from the magnetic field and placed on the appropriate collection tube. CD138-positive cells were washed out with the plunger and collected. After sorting with CD138 magnetic beads, non-CD138-positive cells were collected. RNA was immediately extracted from the sorted MM cells, and total RNA was extracted from the cell samples using a TRIzol reagent and frozen stored at − 80°C. The 20 samples (10 CD138-positive cells and 10 CD138-negative cells) were sequenced, and all data were divided into two groups (positive and negative groups) for analysis. Library construction and sequencing were performed by Annoroad Gene Technology (Beijing, China). All participants signed the informed consent form, and the study was approved by the institutional ethical review board of the Chao-Yang Hospital, Capital Medical University (Beijing, China).

### RNA Sequencing and Identification of DE lncRNAs

After downloading the reference genomes and the annotation file from the ENSEMBL database^1^, the sequencing reads were mapped to reference genomes. The clean data were mapped to the reference genome using HISAT2^2^. The gene expression read count for each gene was quantitated in each sample by HTSeq^3^, and fragments per kilobase million mapped reads (FPKM) were then calculated to represent the expression level of genes in each sample. DESeq2^4^ was used for the differential expression analysis of two groups (CD138 + and CD138 − groups). Novel lncRNA was screened through a variety of coding potential analysis software, including CNCI analysis (Coding–Non-coding index), CPC analysis (Coding Potential Calculator), PFAM protein domain analysis, and CPAT analysis (Coding Potential Assessment Tool). The four analysis methods all judged that the non-coding transcript was the final novel lncRNA data set. Considering that the number of reads coming from a gene (or transcript isoform) follows a binomial distribution, DESeq2 was proposed to standardize data using the negative binomial distribution statistical method. A *P*-value was allotted to each gene and adjusted by the Benjamini and Hochberg (BH) method. The DE genes were identified as genes with *q* ≤ 0.05 and | log2_ratio| ≥ 1. Moreover, 22,923 lncRNAs could be detected by this annotation, including the following biotypes: lincRNA (long intergenic non-coding RNAs), antisense, bidirectional promoter lncRNA, sense overlapping, sense intronic, and 3′ overlapping ncRNA. A total of 9,540 lncRNAs were entered into the database after expression filtering. A protein–protein interaction (PPI) network was constructed by extracting the subnetwork, which consisted of DE protein-coding genes or the *trans* or *cis* targets of DE lncRNAs, from the whole PPI network of corresponding species by directly mapping these genes or targets to the PPI network. The subnetwork file was imported into Cytoscape, and the network was visualized based on the attribute of each gene. Finally, target prediction was carried out, and the mRNAs with high Spearman correlation coefficient (*P* ≥ 0.9) were selected as the *trans* targets. Subsequently, RNAs with a distance less than 50 kb were selected as the *cis* targets.

### RNA Sequencing and Identification of DE miRNAs

The reference genome index was built using Bowtie1, and then the clean reads were mapped to the genome. The reads were mapped to mature miRNA and hairpin, which were recorded in miRBase (release 21), to identify known miRNAs. The rest of the reads were used to predict a novel miRNA after excluding the reads mapped to the known miRNA/ncRNA/repeat region/mRNA region. The miRDeep2 software was used for identification and prediction. For quantization of miRNA expression, transcripts per million were used for assessment and standardization after obtaining the count of mature miRNAs in miRDeep2. Further, DESeq2 was used for differential expression analysis. A *P*-value was assigned to each gene and adjusted by the BH approach for controlling the false discovery rate, assuming that the number of reads derived from an miRNA followed a binomial distribution. miRNAs with *q* < 0.05 and log2 ratio ≥ 1 were identified as DE miRNAs. For miRNA target prediction, target genes of known or novel miRNAs were predicted using MiRanda, PITA (target-site accessibility and free predictors of microRNA targets), and TargetScan.

### RNA Sequencing and Identification of DE circRNAs

CircRNA is mainly exon-cyclized ecircRNA, and its ring-forming mechanism has two models: lariat-driven circularization and intron-pairing-driven circularization. Sequencing reads were mapped to reference genomes. The reference genomes and annotation file were downloaded from the ENSEMBL database (see text footnote 1). The reads were mapped to the reference genome using the BWA-MEM algorithm to split and compare the sequences and then scan the resulting SAM files to find PCC (paired chiastic clipping) and PEM (paired-end mapping) sites, as well as GT-AG splicing signals, and finally will have the sequence of the junction site which was realigned with a dynamic programming algorithm, and this method was used for identification. Differential expression analysis was performed by DESeq2 (see text footnote 4). Assuming that the number of reads coming from a gene (or transcript isoform) follows a binomial distribution, DESeq2 was used for differential expression analysis based on the MA plot. A *P*-value could be assigned to each gene and adjusted by the BH method. DE genes were identified as genes with *q* ≤ 0.05 and | log2_ratio| ≥ 1. Finally, sponge prediction was carried out, and the target circRNA of miRNA was predicted by MiRanda.

### Enrichment Analysis of Gene Ontology and Kyoto Encyclopedia of Genes and Genomes Pathway

Gene Ontology (GO) provides a dynamically updated set of standard vocabulary (control vocabulary) to depict the nature of genes completely as an international standardized classification system of gene function. GO has three ontologies: molecular function, cell component, and biological process (BP) of genes. To investigate whether genes are from one GO (Gene Ontology)^5^ term, a hypergeometric *p*-value is calculated and adjusted as *q*-value, where the background is set to be genes in the whole genome. GO terms with *q* < 0.05 are considered to be significantly enriched. GO enrichment analysis shows what biological functions the DEGs perform.

The Kyoto Encyclopedia of Genes and Genomes (KEGG)^6^ contains a collection of manually drawn pathway maps representing proteins and gene products responsible for molecular interaction and reaction networks. KEGG (Kyoto Encyclopedia of Genes and Genomes see text footnote 6) is a database resource which contains a collection of manually drawn pathway maps representing our knowledge on the molecular interaction and reaction networks. Using the same method with GO enrichment analysis, significantly enriched KEGG pathways are identified.

Significant enrichment of ncRNAs was found using KEGG pathway and GO enrichment analyses. Terms with *q* < 0.05 were considered to be significantly enriched.

### ceRNA Sequencing

CeRNA includes lncRNA–mRNA, microRNA–lncRNA, microRNA–mRNA, and cicrRNA–microRNA functional analyses and ternary network analysis. Whole-transcriptome association analysis is a deep analysis based on the standard analysis results of mRNA, lncRNA, circRNA, and microRNA. The results of gene differential expression analysis and microRNA target prediction in the standard analysis were used to comprehensively study the regulation of non-coding RNA on mRNA expression.

Pre-miRNA Identification and miRNA Target Prediction: an lncRNA regulatory network was constructed to reveal the role and interactions among lncRNAs and miRNAs in MM pathogenesis. MiRDeep2 was used to identify the pre-miRNAs in the lncRNA cluster. Target genes of known or novel miRNAs were predicted by MiRanda, PITA, and TargetScan, and the target genes were the cumulative outcome of the prediction using at least two programs.

Analysis of ceRNA by the enrichment of GO and KEGG: The GO and KEGG enrichment of miRNA target genes was performed using the hypergeometric test, in which the *P*-value was calculated and adjusted as the *q*-value, and the background data were genes in the whole genome. The GO and KEGG terms with *q* < 0.05 were considered to be significantly enriched.

### Quantitative Real-Time Reverse Transcriptase–PCR

The reverse transcription (RT) reaction system was prepared as follows. The RT reaction (volume 20 μL) conditions were set as 42°C for 50 min and 85°C for 5 min. After the reaction, the solution on the wall of the tube was collected at the bottom of the tube by centrifugation for a short time, and the RT product of the DNA was stored at − 20°C. The amplification of lncRNA was conducted with GenePool (Cat# GPQ1808) using an RNA/lncRNA quantitative polymerase chain reaction (qPCR) kit. The experimental operation was carried out according to the product instructions. The components in the kit were dissolved and put on ice for reserve. The components were added to the reaction tube (dNTP Mix, 2.5 mM; RNase-Free Water 4 μL; Primer Mix, 2 μL; RNA Template 2 μL; 5 × RT buffer, 4 μL; DTT, 0.1 M × 2 μL; HiFiScript, 200 U/μL × 1 μL; RNase-free water up to 20 μL) and mixed. The total volume was 20 μL. They were incubated at 42°C for 50 min and at 85°C for 5 min. After the reaction, the solution on the wall of the tube was collected at the bottom of the tube by centrifugation for a short time, and the RT of the DNA was stored at − 20°C. Fluorescence qPCR amplification conditions were set as follows: 95°C for 30 min; 95°C for 5 s, and 60°C for 30 s for 45 cycles. The amplification of miRNA was performed using an miRNA qPCR kit (GenePool, Cat # GPQ1809). The amplification conditions were set as follows: 95°C for 30 s, 95°C for 5 s, and 60°C for 30 s for 45 cycles.

## Results

### Clinical Characteristics of Patients With MM

All NDMM (newly diagnosed multiple myeloma) diagnoses were confirmed. The clinical and genetic characteristics of each patient were recorded. The clinical characteristics of the 10 patients with MM are shown in Supplementary Table 1.

### Expression Distribution Statistics

**FIGURE 1:**
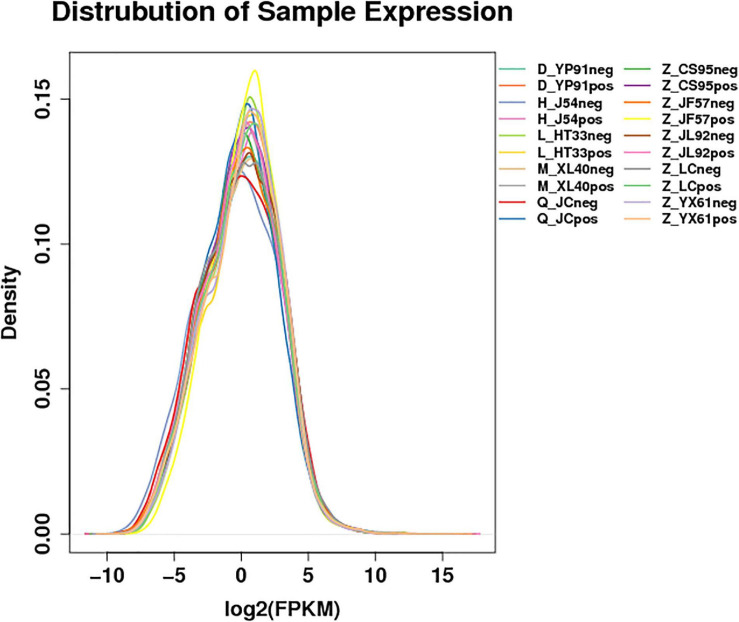
Expression profile of lncRNAs. Distribution map of the expression profile: For the gene expression of each group of samples, the density distribution was made after taking the logarithm of base 2. The horizontal coordinate was log2 (FPKM + 0.0001), and the ordinate was the density of genes. Different colors represent different samples.

**FIGURE 2:**
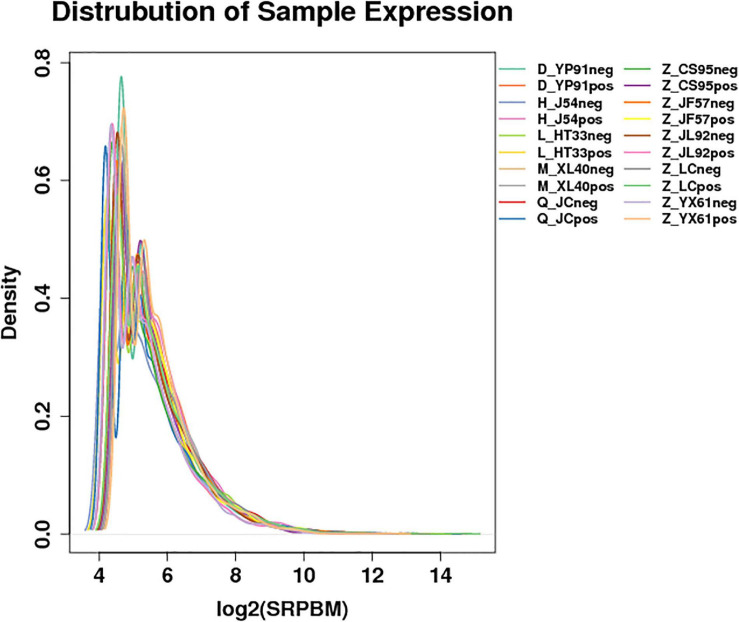
Expression of the density map of circRNAs: For the gene expression of each group of samples, the density distribution was made after taking the logarithm of base 2. The horizontal coordinate was log2 (SRPBM), and the ordinate was the density of genes. Different colors represent different samples.

According to the gene expression of all samples, the expression density map of the lncRNA was created as shown in Figure 1. The overall distribution trend of the expression of the samples was observed. According to the position relationship between lncRNA and coding sequence, lncRNA can be divided into lincRNA (long intergenic non-coding RNAs), intronic lncRNA (intronic transcript lncRNA), antisense lncRNA, sense lncRNA, and bidirectional lncRNA. After being screened, 22923 lncRNAs, 2094 microRNAs, and 22566 circRNAs were obtained. The expression density map of the circRNA is shown in Figure 2.

### DE (Differentially Expressed) ncRNAs and mRNAs

**FIGURE 3:**
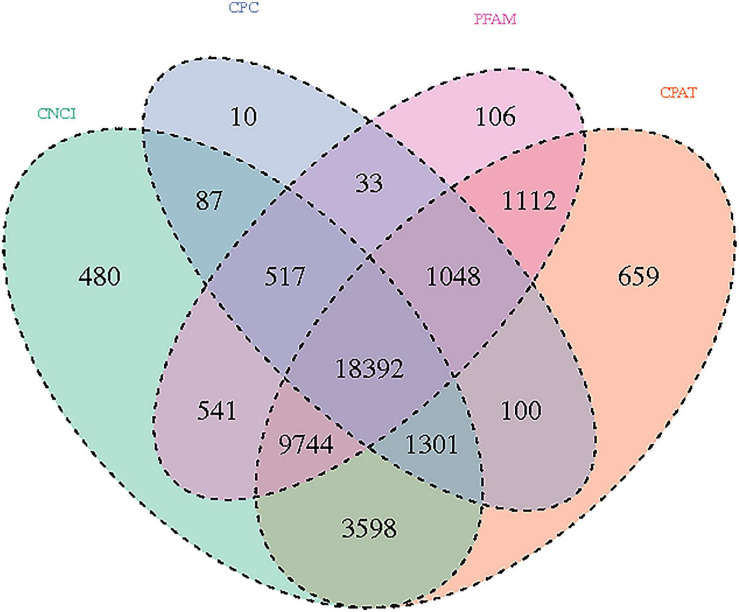
Novel lncRNAs: Venn diagram results using different software prediction tools, including coding–non-coding index (CNCI), coding potential calculator, protein folding domain database (PFAM), and coding potential assessing tool software, showing upregulated and downregulated lncRNAs whose dysregulated expression pattern was shared by 10 patients with MM.

**FIGURE 4:**
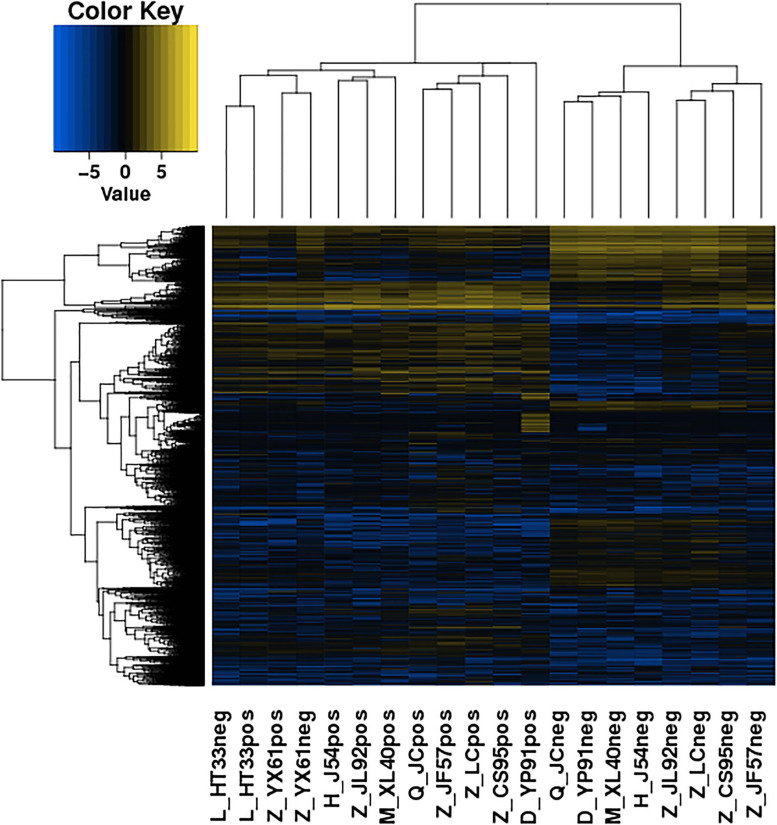
Heatmaps of DE lncRNA: According to the expression of DE genes in each sample, the logarithm of base 2 was considered, the Euclidean distance was calculated, the system clustering method (hierarchical cluster) was used, and the clustering results of the sample were obtained. The various colors represent different expression levels. Blue color indicates the lower expression level, and yellow color indicates the higher expression levels.

**FIGURE 5:**
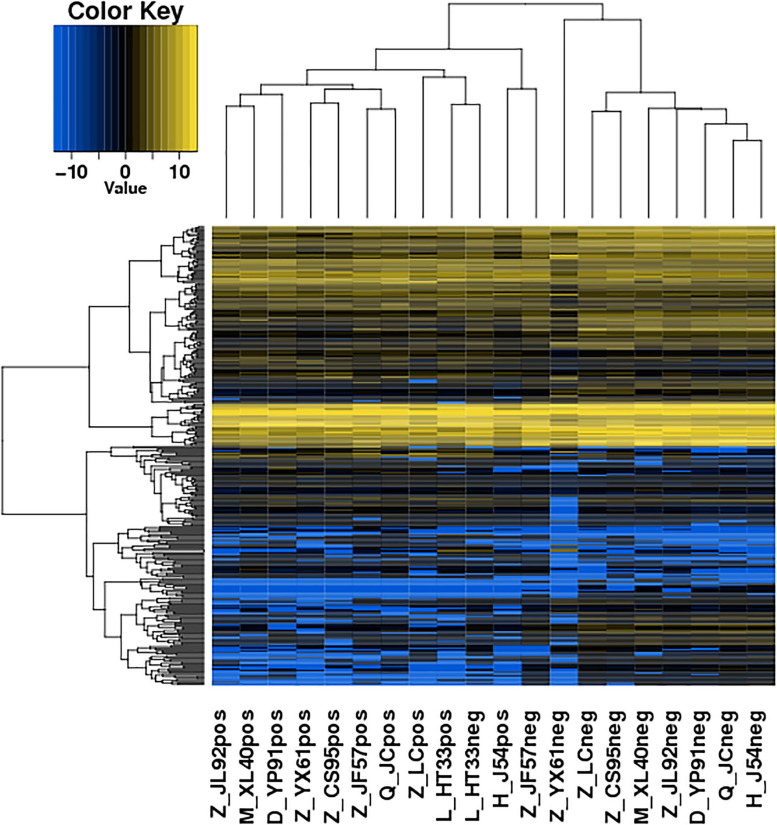
Heatmaps of DE microRNA: According to the expression level of differentially expressed microRNA in each sample, the clustering results of the sample were obtained. In the figure, the various colors represent different expression levels. Blue color indicates the lower expression level, and yellow color indicates the higher expression levels.

**FIGURE 6:**
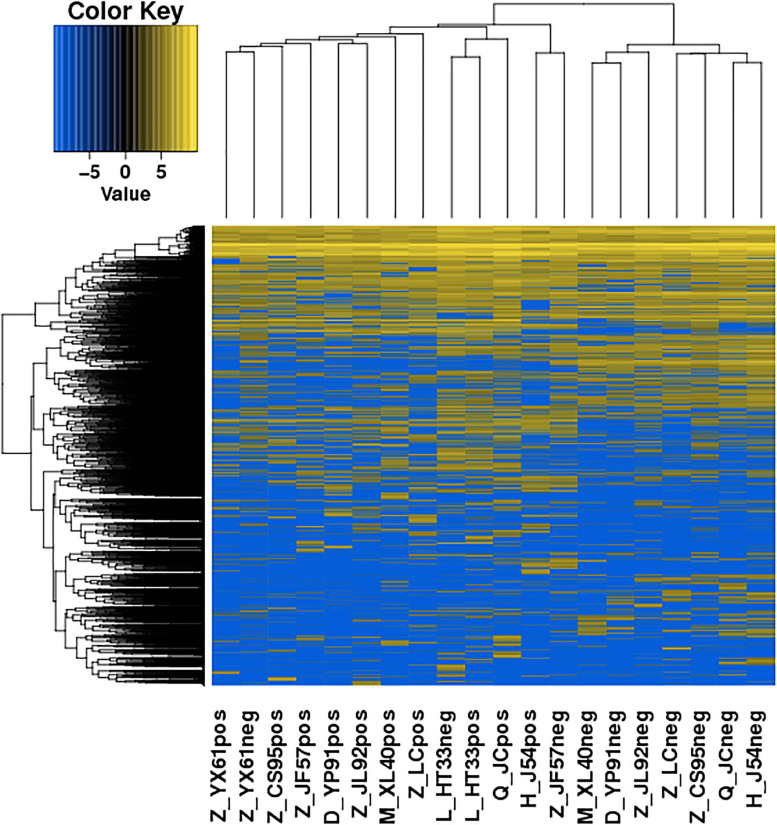
Heatmaps of DE circRNA: According to the expression level of differentially expressed circRNA in each sample, the clustering results of the sample were obtained. In the figure, the various colors represent different expression levels. Blue color indicates the lower expression level, and yellow color indicates the higher expression levels.

The significance of DE lncRNAs, miRNA, circRNA, and mRNAs was analyzed by the deep sequencing based on the Illumina sequencing platform. Compared with CD138-negative cells, a total of 4,419 DE lncRNAs (1,559 known lncRNAs and 2,860 novel lncRNAs; among them, 2,370 genes upregulated and 2,049 genes downregulated), 355 DE miRNAs (340 known miRNAs and 15 novel miRNAs), and 1,044 DE circRNAs (527 upregulated genes and 517 downregulated genes) were significantly regulated in the malignant plasma cells (CD138-positive cells) of 10 patients. Furthermore, a total of 5,522 DE mRNAs were identified in MM, in which 2,091 were upregulated and 3,431 were downregulated. We assembled transcripts by stringTie software before analyzing their coding potential for the identification of new lncRNAs. First, each sample was spliced separately and then combined with stringTie merge. stringTie software divided the reads into different classes at first and then generated a mosaic map for each class to determine the transcripts. Each transcript generated a maximum flow algorithm to evaluate the expression level and assemble the complex data into transcripts. Three types of lncRNA, including lincRNA, intronic lncRNA, and antisense lncRNA, were selected to screen novel lncRNA. Screening is carried out through a comprehensive variety of coding potential analysis software, mainly through CNCI (Coding–Non-coding Index), CPC (Coding Potential Calculator), PFAM protein domain analysis, and CPAT analysis. The four analysis methods all judged that the non-coding transcript was the final novel lncRNA data set. The non-coding transcripts identified by the above methods were counted, and the common and unique numbers of each method were shown by the Venn diagram. The Venn diagram of novel lncRNAs of the 10 patients with MM is shown in Figure 3. CIRI is an efficient and fast circular RNA identification tool. First, use the BWA-MEM algorithm to split and compare the sequences, and then scan the resulting SAM files to find PCC (paired chiastic clipping) and PEM (paired-end mapping) sites, as well as GT-AG splicing signals, and finally have the sequence of the junction site which was realigned with a dynamic programming algorithm. The results of differentially expressed circRNAs are all novel. The clustering results of the ncRNA of the samples according to the expression of differentially expressed genes in each sample are shown in Figure 4 (heatmap plot of differentially expressed of lncRNAs), Figure 5 (heatmap plot of differentially expressed of microRNAs), and Figure 6 (heatmap plot of differentially expressed of circRNAs).

### Functional Prediction of DE ncRNAs

Further, the function of all DE ncRNAs was predicted using analyses of GO and KEGG pathways to examine the function of ncRNAs in MM. For GO/KEGG of ncRNAs, sequence annotation gene.fa documents were obtained by extracting the gene sequence according to the location of the gtf file, but not obtained directly from the annotation file; the comment information is found through blast by using the gene.fa file. In the results of lncRNAs and miRNAs, the target gene mRNA was annotated. GO enrichment analysis and KEGG pathway analysis were performed for the target genes of differentially expressed ncRNAs, including known and novel ncRNAs.

**FIGURE 7:**
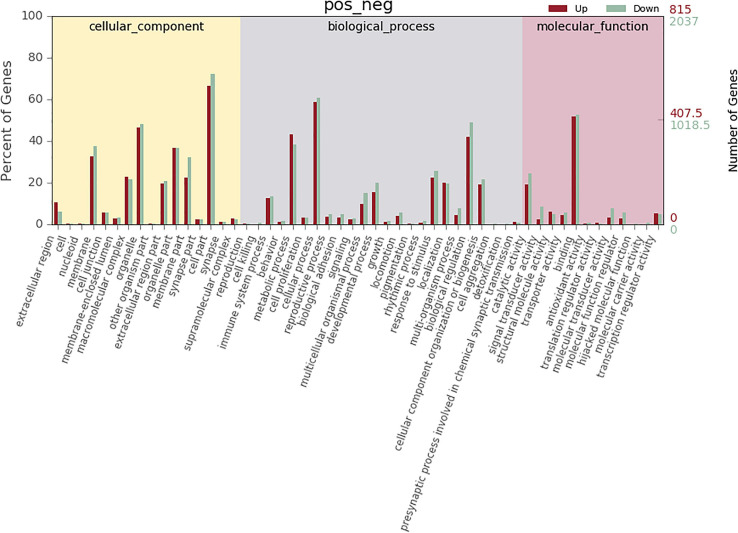
GO analysis of lncRNA in MM. The significant molecular function, biological process, and cellular component changed mRNA-targeted ncRNAs in MM. Statistics of DE gene annotation results in GO entries. The abscissa is the second-level GO entry in the DE gene annotation results, the left ordinate is the proportion of upregulated/downregulated DE genes, and the right ordinate is the number of upregulated/downregulated DE genes.

**FIGURE 8:**
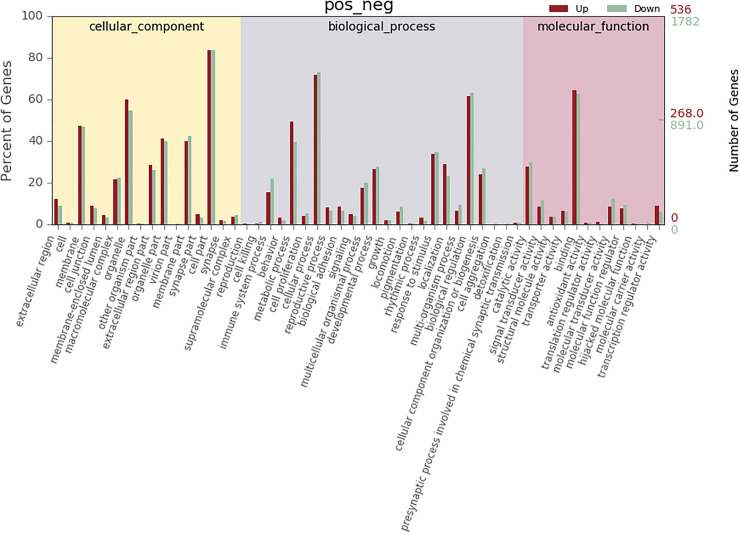
GO analysis of microRNA in MM. The significant molecular function, biological process, and cellular component changed mRNA-targeted ncRNAs in MM. Statistics of DE gene annotation results in GO entries. The abscissa is the second-level GO entry in the DE gene annotation results, the left ordinate is the proportion of upregulated/downregulated DE genes, and the right ordinate is the number of upregulated/downregulated DE genes.

According to co-location and co-expression of DE lncRNAs and miRNAs based on GO analysis, the most significant enrichment in BP (biological process) was due to regulation of cellular process, biological regulation, and regulation of biological process, and the most significant enrichment in CC (cellular component) was due to cell part, organelle, and membrane. Binding and catalytic activity were most significantly enriched in MF (molecular function). The results of lncRNAs are shown in Figure 7; miRNAs are shown in Figure 8.

According to GO analysis of DE circRNAs, the most significant enrichment in BP was due to B cell receptor signaling pathway, CD4-positive and alpha-beta T cell activation, DNA alkylation, and DNA conformation change; the most significant enrichment in CC was due to AP-type membrane coat adaptor complex, ATPase complex, BRCA1-A complex, CAAX–protein geranylgeranyltransferase complex, and DNA-dependent protein kinase–DNA ligase 4 complex; and the most significant enrichment in MF was due to 1-phosphatidylinosital-3-kinase activity, 1-phosphatidylinosital-4-phosphate 5-kinase activity, ATP binding, and ATP-dependent DNA helicase activity.

**FIGURE 9:**
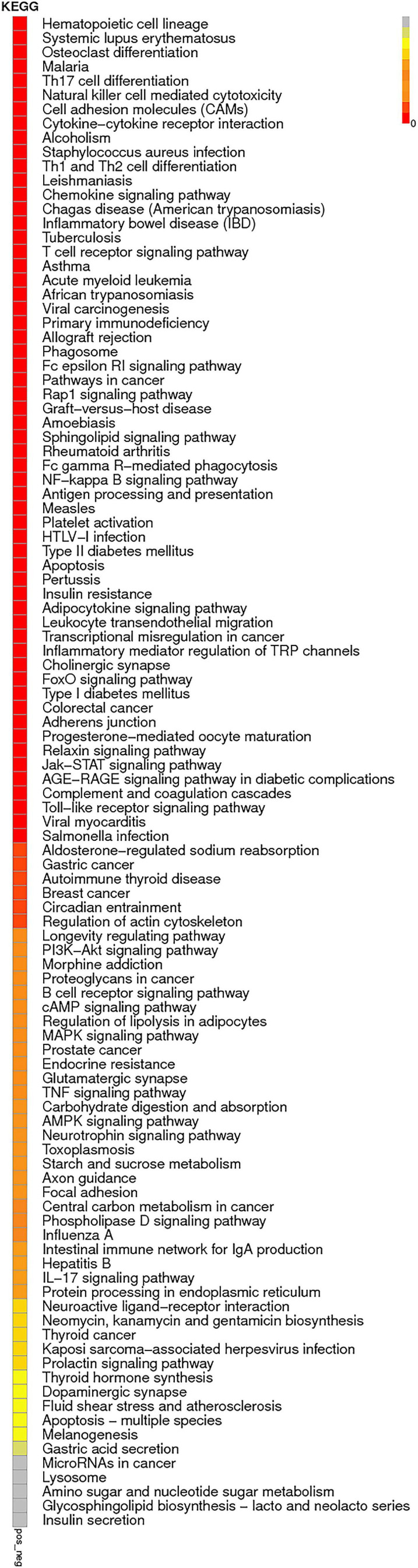
KEGG pathway of lncRNAs:KEGG pathway scatterplots in the plasma cells of MM. The KO enrichment of all samples was combined, and the distribution map was drawn according to the significant *Q*-value of the enrichment of the samples. Each point indicates the degree of enrichment of the KO entry, and the closer the color approaches red, the higher the degree of enrichment is. The size of each point indicates the number of genes enriched in the KO entry. The larger the point is, the more the genes are enriched in the KO entry, and vice versa.

**FIGURE 10:**
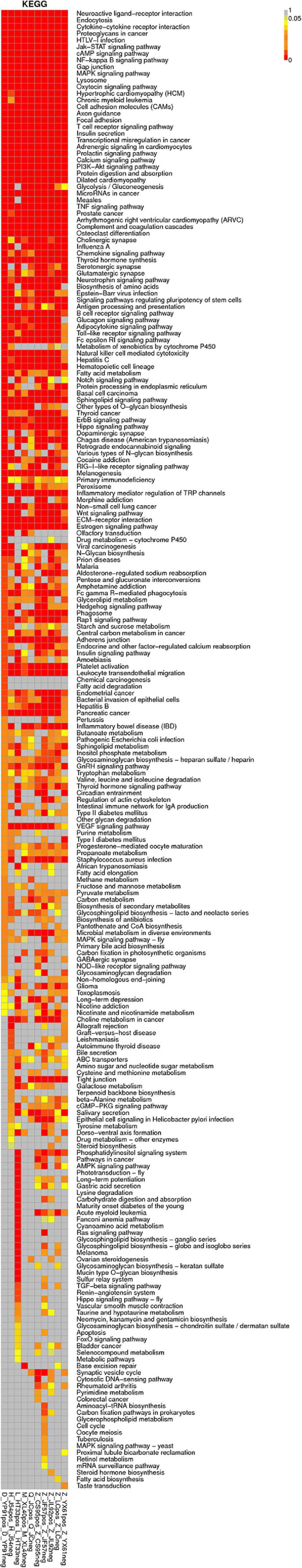
KEGG pathway of microRNA.

**FIGURE 11:**
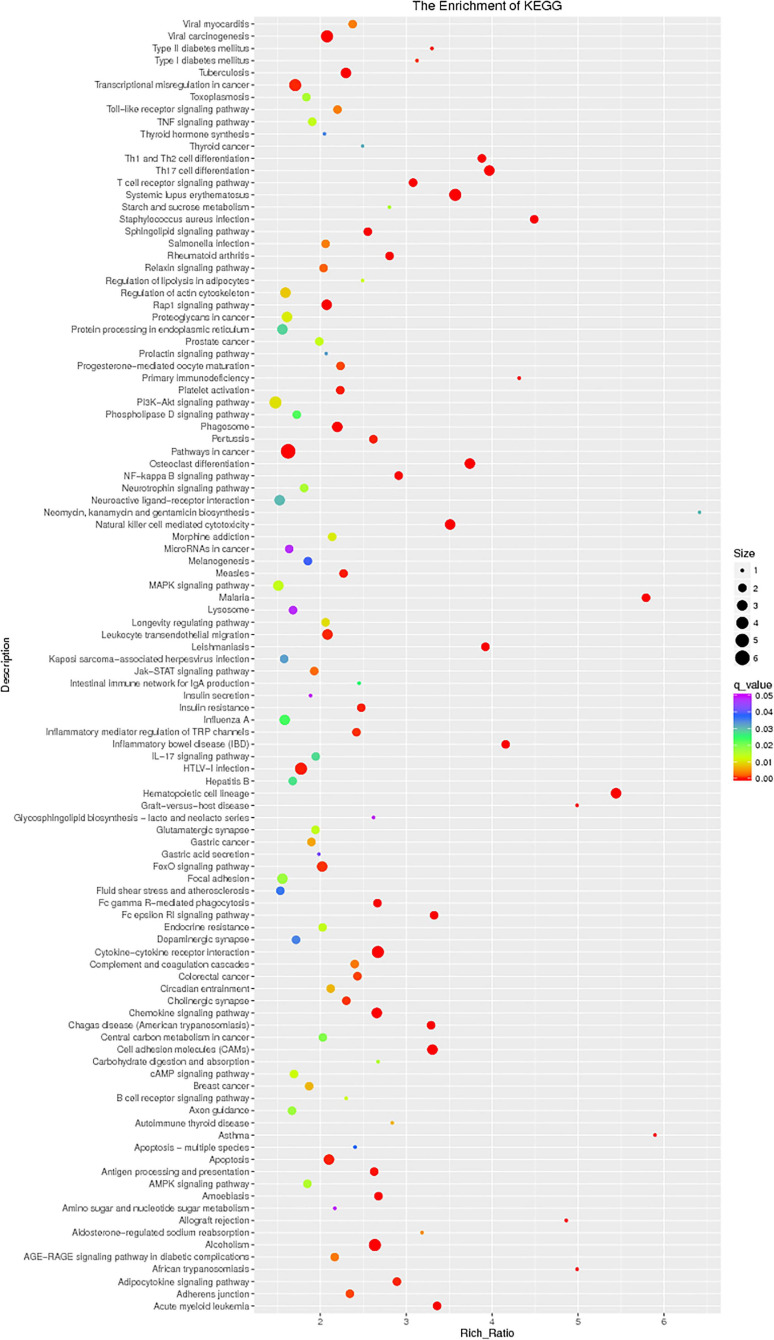
KEGG pathway of circRNA.

The enrichment analysis of each KEGG pathway was carried out using the hypergeometric test to determine the significant enrichment pathway of DE genes. The KEGG pathway of lncRNAs is shown in Figure 9; the most significantly enriched pathways included hematopoietic cell lineage, osteoclast differentiation, chemokine signaling pathway, inflammatory mediator regulation of TRP channels, NOD-like receptor signaling pathway, relaxin signaling pathway, phagosome, Th17 cell differentiation, apoptosis, and NF-kappa B signaling pathway. miRNAs of the KEGG pathway are shown in Figure 10; the most significantly enriched pathways included neuroactive ligand–receptor interaction, endocytosis, cytokine–cytokine receptor interaction, proteoglycans in cancer, jak-STAT signaling pathway, NF-kappa B pathway, and MAPK signaling pathway. circRNAs of the KEGG pathway are shown in Figure 11; viral carcinogenesis, transcriptional misregulation in cancer, Th17 cell differentiation, Rap signaling pathway, and cytokine–cytokine receptor interaction were the top enriched pathways in circRNA.

The most significant enrichment based on the GO analysis of targeted genes of DE lncRNAs, target genes of DE miRNAs, and predicted mRNAs was the same as the enrichment obtained based on the colocalization and co-expression of genes of DE lncRNAs, target genes of DE miRNAs, and predicted mRNAs.

### qPCR Verification of lncRNAs

The expression of some DE lncRNAs and DE miRNAs was analyzed, and the reliability of the sequencing results was verified, thus providing the basis for further studies. According to sequencing results, six differentially expressed known lncRNAs and six novel lncRNAs were selected and verified by Q-PCR (Supplementary Table 2). At the same time, three related miRNAs (hsa-miR-345-5p, hsa-miR-193b-3p, hsa-miR-338-5p)were selected and verified by qPCR based on these 12 lncRNAs (Supplementary Table 3). We found that the qPCR and sequencing results of four known lncRNA (MIAT, KCNQ1OT1, A2M-AS1, and CTA-292E10.6) and five novel lncRNA (MSTRG.155519, MSTRG.190620, MSTRG.193521, MSTRG.260088, and MSTRG.13132) were consistent and can be verified.

**FIGURE 12:**
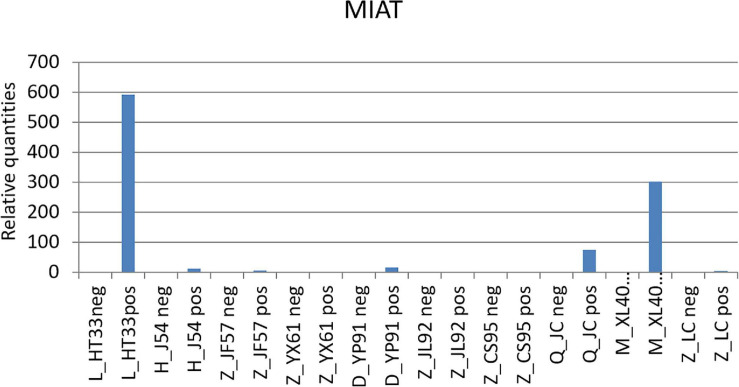
qPCR results of MIAT. MIAT was upregulated in the patients with MM. According to the original detection results of RT-PCR, and the relative quantities was calculated by 2–△△ct, the difference of the transcription level of MIAT of each sample was calculated. The ordinate is the relative expression of MIAT in MM.

Among these known lncRNAs, the myocardial infarction–associated transcript (MIAT) was found upregulated (gene location: chr22: 26646428–26676475: +, Log2 fold change 1.583265774, p0.0002, primer ACCTTGACTAACTCCTGCCTTC, product length 276) in previous studies on various tumors, but was not found participated in the pathological and physiological processes of MM (Luan et al., 2017). The results of the qPCR are shown in Figure 12.

### Carcinogenic Pathway and lncRNAs

**FIGURE 13:**
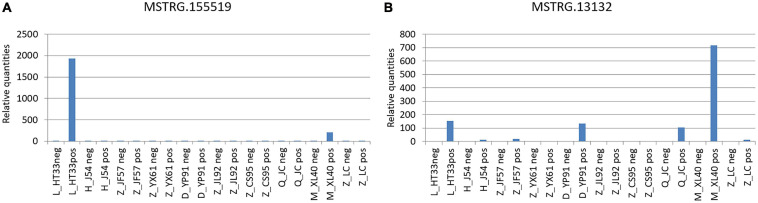
Verification of novel lncRNAs: According to the original detection results of RT-PCR, and the relative quantities calculated by 2−△△ct, the difference of the transcription level of target gene of each sample was calculated. The ordinate is the relative expression of novel lncRNA in MM. **(A)** MSTRG.155519 performed by qPCR; **(B)** MSTRG.13132 performed by qPCR.

Prediction of the correlation between lncRNA and MM pathogenic genes from the oncogene pathway using lncRNA *cis* target and lnc_TransTar.xls lncRNA *trans* target revealed two novel DE lncRNAs among the following verifiable genes: NRAS, FAM46C, CXCR4, PIK3CA, ATP13A4, FGFR3, WHSC1, FAT1, PRDM9, IL7R, PDGFRB, HLA-B, PRDM1, IKZF1, EGFR, BRAF, KDM6A, MYC, CCND1, BIRC3, BIRC2, ATM, PTPN11, RB1, DIS3, TRAF3, CYLD, TP53, SAMHD1, MAFB, carcinoembryonic antigen-related cell adhesion molecule 1 (CEACAM1), IGLL5, and XBP1 (Kortüm et al., 2015; Kortuem et al., 2016; Lionetti and Neri, 2017; Bolli et al., 2018). This was verified using qPCR (Figure 13).

(1) MSTRG.155519 and CEACAM1: The CEACAM1 was the downstream pathway of MSTRG.155519 through the prediction of lnc_TransTar.xls lncRNA.
(2) MSTRG.13132 and FAM46C: FAM46C was the downstream pathway of MSTRG.13132 through the prediction of the lncRNA *cis* target.

### Analysis of the Regulatory Network of ncRNAs and mRNAs (ceRNAs)

The ceRNA theory proved that lncRNA had an extensive regulatory function, acting as a sponge and competitive binding site of small RNA. In this study, the regulatory network of ncRNAs and mRNAs was analyzed to explore the molecular mechanism of ncRNAs. The standardized analysis of mRNA, lncRNA, circRNA, and miRNA can be achieved by the construction of specific transcriptome library and sRNA library, and the function of lncRNA can be predicted indirectly. According to these results, we can get the differential expression of lncRNA and its target mRNA. Target mRNA and target lncRNA with the same miRNA binding site were found.

**FIGURE 14:**
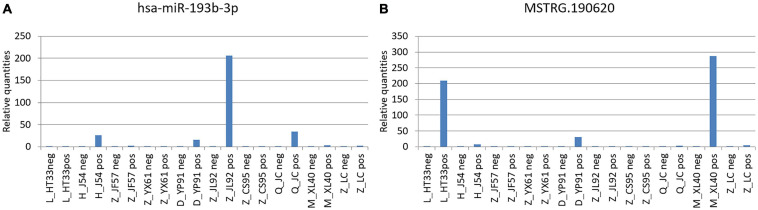
Novel lncRNA MSTRG.190620 and miR-193b-3p constituted ceRNA of lncRNA–miRNA–mRNA. **(A)** miR-193b-3p was verified by PCR in MM. **(B)** Novel lncRNA MSTRG.190620 was verified by PCR in MM. The difference of the transcription level of target gene of each sample was calculated by 2–△△ct. The ordinate is the relative expression of the target gene in MM.

In these miRNAs, it was speculated that some pairs of ceRNAs (lncRNA–miRNA–mRNA) were found using sequence analysis. Among these, novel lncRNA MSTRG.190620 and miR-193b-3p, which constituted ceRNA of lncRNA–miRNA–mRNA, were identified (Figure 14). MiR-193b-3p and MSTRG.190620 were upregulated in malignant plasma cells. The abnormal expression of hsa-miR-193b has been proved to be one of the pathogeneses of malignant tumors (Gonzalez-Gugel et al., 2013; Li et al., 2017).

## Discussion

MM is a heterogeneous malignant hematological tumor caused by the abnormal proliferation of malignant plasma cells in bone marrow. The occurrence, development, and prognosis of MM are driven by different genetic events. Although the therapeutic agents of MM have improved in the last 10 years, it is still incurable in most cases. Previous studies of functional genomics and epigenomics showed that, similar to protein-coding genes, ncRNAs were dysregulated which played a crucial role in the pathogenesis of human malignant tumors (Garzon et al., 2010; Amodio et al., 2017), including MM (Ahmad et al., 2014). miRNAs have been extensively studied. Notably, lncRNAs which transcribed over half of the human genome deserve more attention at present. lncRNAs are non-coding transcripts of more than 200 nucleotides in length (Johnsson et al., 2014).

In this study, ncRNAs were reported as an important genomic component with a significant impact on the biological behavior of MM. Among ncRNAs, more attention has been paid to lncRNAs. lncRNA is confirmed to regulate gene transcription and mRNA translation through a variety of mechanisms, including epigenetic modification of gene expression, regulation of interaction with RNA-binding proteins, and regulation of miRNA (Wang and Chang, 2011). Therefore, lncRNA is likely to be a tumor-related gene, acting as oncogene or tumor suppressor (Ling et al., 2015), and may become a potential target of therapy and provide the basis for the development of new drugs (Chandra Gupta and Nandan Tripathi, 2017). The role of previously studied lncRNAs, such as homeobox transcriptional antisense RNA (HOTAIR) and metastasis-associated lung adenocarcinoma (MALAT1), maternally expressed gene 3 (MEG3), taurine upregulated1 (TUG1), and nuclear paraspeckle assembly transcript 1 (NEAT1) (Zhou et al., 2007; Benetatos et al., 2008; Cho et al., 2014; Isin et al., 2014; Handa et al., 2017; Amodio et al., 2018; Dahai et al., 2019; Taiana et al., 2019; Shehata et al., 2020), have been widely accepted for their biological functions and mechanisms in the development of MM. However, the precise biological role of the majority of lncRNAs remains unclear. This study provides a rationale for further detailed functional exploration of lncRNAs and their impact on MM biology, focusing on the potential therapeutic application in MM. Further, more novel lncRNAs should be explored, and the functional and regulatory interactions between novel lncRNAs and miRNAs in MM should be predicted. This study investigated DE ncRNAs more broadly and also preliminarily discussed the expression of circRNA in MM. In this study, new MM cases were selected to detect ncRNAs. Plasma cell sorting was performed in all patients, and the differential expression was found to be more accurate than that in most small-sample studies. Therefore, this study investigated DE ncRNAs more broadly and predicted the functional and regulatory interactions between novel lncRNAs and miRNAs in MM cells. The expression of circRNA in MM cells was also discussed preliminarily.

This study concluded that lncRNAs and miRNAs were significantly dysregulated in MM cells. A total of 4,419 DE lncRNAs (1,559 known lncRNAs and 2,860 novel lncRNAs), 355 DE miRNAs (340 known miRNAs and 15 novel miRNAs), and 1,044 DE circRNAs (527 genes were upregulated and 517 genes were downregulated) were significantly regulated in malignant plasma cells of NDMM. In this study, more new lncRNAs were detected, indicating the lack of preliminary studies on the function and role of lncRNAs in MM. Further, the results obtained through the whole-genome sequencing study were more comprehensively compared with the findings of the gene chip technology. Although several lncRNAs were found deregulated in plasma cell dyscrasia, only a few have been functionally validated in biological and disease processes. Among these genes, MIAT was overexpressed in malignant plasma cells. MIAT is a subnuclear lncRNA associated with a variety of high-risk heart diseases, such as acute myocardial infarction, and has been reported to be associated with nervous system neoplasm. In the present study, MIAT was confirmed to be crucial in the growth of tumor cells, invasion, and promotion of apoptosis of tumor cells. A recent study on colorectal cancer and MIAT showed that MIAT promoted the growth and metastasis of colorectal cancer cells by regulating the Mir-132/Derlin-1 pathway, indicating the ceRNA relationship between MIAT and Mir-132, which proved that MIAT played the role of ceRNA in Mir-132 (Liu et al., 2018). Recent studies confirmed that MIAT knockout eradicated the extended migration and survival of neuroblastoma and glioblastoma cell lines and increased the apoptosis of basal cells (Bountali et al., 2019). Although the mechanism underlying this deregulation and its significance in tumorigenesis are still poorly understood, MIAT may still become a potential therapeutic target for MM. Further experiments should be conducted *in vivo* and *in vitro* in the future.

The expression profile based on molecular characteristics has become an increasingly important and powerful prognostic tool to predict outcomes in patients with MM. This study intended to predict the relationship between lncRNA and MM pathogenic genes through the oncogene pathway by targeting 33 oncogenes and suppressor genes that have been published and confirmed to be related to the pathogenesis of MM. Two parts of the ncRNAs were obtained, including known genes and novel genes, through high-throughput sequencing. In this study, the novel genes were focused to further clarify the biological characteristics of ncRNAs in MM. Target genes and DE lncRNAs were analyzed using *cis* and *trans* target prediction, respectively. Among these, two novel lncRNAs were selected and verified by PCR. These two novel lncRNAs had no annotations in the existing database. Two new lncRNAs were found to be associated with oncogenes: MSTRG.155519, a new upstream molecule in the pathway of MM, and CEACAM1, the downstream regulatory gene of MSTRG.155519, which was found through the prediction of lnc_TransTar.xlslncRNA.CEACAM1, known as CD66a (cluster of differentiation 66a), are members of the carcinoembryonic antigen family and the only CD66 abnormally expressed on the surface of plasmacytes. Members of the immunoglobulin superfamily assume a double status in malignancies (Calinescu et al., 2018). Previous researches have shown a higher expression of CEACM1 in MM. Extramedullary lesions were associated with CEACAM1 (Josef et al., 2010; Guinn et al., 2011; Johnson and Mahadevan, 2015; Xu et al., 2018). The other novel lncRNA was MSTRG.13132, related to FAM46C, the downstream pathway of MSTRG.13132. Partial deletion of the short arm (1p) of chromosome FAM46C:1 frequently occurs in MM. The common segments are 1 p32.3, 1 p31.3, 1 p22.1–1 p21.3, and 1 p12. Among these, homozygous deletion of 1 p12 (FAM46C) is one of the most valuable areas for research and may act as a tumor inhibitor in MM (Mroczek et al., 2017; Zhu et al., 2017; Ryland et al., 2018; Walker et al., 2018). Previous studies found that wild-type FAM46C could induce growth inhibition and apoptosis of MM cells. The viability of the cells decreased by 50–80% in 6 days in MM cell lines. The overexpression of FAM46C shortened the survival time of MM cells by downregulating the expression of interferon regulatory factor-4 and MYC (Zhu et al., 2017). These studies confirmed that the expression of FAM46C could induce MM growth inhibition and apoptosis and was crucial in the occurrence, development, and apoptosis of MM. lncRNAs, which regulate these MM oncogenes, could be potential therapeutic targets for MM. A recent study analyzed the expression profiles of lncRNAs in MM by reusing the publicly available microarray data exposed in the database. Bioinformatics analysis was used to predict the biological functions of prognostic lncRNAs (Zhou et al., 2015). In our study, the expression of ncRNA was monitored by sequencing, and the relationship between oncogenes and lncRNAs was obtained by analyzing the data in *cis* and *trans* forms.

At present, lncRNA and circRNA have been found to regulate mRNA expression via different mechanisms. A specific transcriptome library and an sRNA library were constructed at the same time to realize the standardized analysis of mRNA, lncRNA, circRNA, and miRNA so as to examine the interactive regulation of various ncRNAs. Besides the analysis of the four types of ncRNAs, the prediction and analysis of ceRNA were carried out. In this study, novel lncRNA MSTRG.190620 was found to build the interaction diagram with hsa-miR-193b-3p. The qPCR results were consistent with the sequencing results of related miRNAs (hsa-miR-193b has been confirmed to be involved in the pathogenesis of malignant tumors) (Wang et al., 2016; Song et al., 2018; Lin et al., 2019). This study speculated using sequence analysis that ceRNA was associated with the lncRNA–miRNA–mRNA network. The results revealed that miR-193b-3p (upregulated in MM) and the novel lncRNA MSTRG.190620 constituted ceRNAs in malignant plasma cell. Hsa-miR-193b-3p was found to be upregulated in different non-viral vectors and could thus be used as a potential target in the non-viral cancer gene treatment (Lin et al., 2019).

In our study, circRNAs were detected. Emerging evidence showed the presence of circRNA with a closed-ring structure in human cells, and endogenous circRNA-regulated gene expression acted as a molecular sponge and thus restrained their function by binding to miRNAs or other molecules. CircRNAs in human cells might be important in the development of tumors. Although the expression of circRNAs was detected in this study, it is still in the early stage of research; the results of our circRNA are all unknown, and little information is found on the annotation of circRNAs. Therefore, the biological functions of these circRNAs in both physiological and pathological processes in MM need to be further explored.

A new data analysis model was proposed in this study due to the predictive role and mutual regulated relationship of lncRNA, miRNA, circRNA, and mRNA in MM pathogenesis. The present study showed that ncRNAs were completely provided by the whole-genome sequencing method. This study also provided a catalog and information of the predicted differential expression of ncRNAs in MM cells and identified several genes associated with the pathogenesis of MM by sequencing, thus contributing to the further exploration of ncRNAs in MM. In this study, the specific expression of ncRNAs in malignant plasma cells was different from the bone marrow stromal cells, indicating that MM cells have different biological characteristics. The next step should be to study proteomics and related signaling pathways of these predicted ncRNAs, which might fully elucidate the underlying mechanism of MM, and ncRNAs might become an index to evaluate MRD.

## Data Availability Statement

The original contributions presented in the study are included in the article/Supplementary Material, further inquiries can be directed to the corresponding author/s.

## Ethics Statement

The studies involving human participants were reviewed and approved by the Chao-Yang Hospital, Capital Medical University (Beijing, China). The patients/participants provided their written informed consent to participate in this study.

## Author Contributions

ML and WC conceptualized this study. ML worked on the methodology, helped in writing and preparing the original draft, and participated in visualization. YW helped in software application and helped in data curation. WG carried out the validation. YT conducted the formal analysis. GW participated in the investigation. AL collected the resources. WC participated in writing, reviewing, and editing, helped in supervision, and carried out the project administration. All authors contributed to the article and approved the submitted version.

## Conflict of Interest

The authors declare that the research was conducted in the absence of any commercial or financial relationships that could be construed as a potential conflict of interest.

## Footnotes

1 http://www.ensembl.org/index.html

2 http://ccb.jhu.edu/software/hisat2/index.shtml

3 http://www-huber.embl.de/users/anders/HTSeq/doc/overview.html

4 http://www.bioconductor.org/packages/release/bioc/html/DESeq.html

5 http://geneontology.org/

6 http://www.kegg.jp/
