# Supplementary material for: Novel Non-coding RNA Analysis in Multiple Myeloma Identified Through High-Throughput Sequencing
Source: Front Genet. 2021 May 24;12:625019. doi: 10.3389/fgene.2021.625019 (PMC8181418; doi:10.3389/fgene.2021.625019)
Supplement: Supplementary file 1 [file Data_Sheet_1.pdf]

Supplementary

Table 1. Clinical characteristics of patients with MM

Characteristics	Patient number (n = 10)
Age (year) ≧60	6（60%）
Sex Male	6（60%）
Female	4（40%）
Immunoglobulin type	
IgA	2（20%）
IgG	7（70%）
Light chain type	1（10%）
International staging system at diagnosis	
DS stage I	1（10%）
II	2（20%）
III	7（70%）
ISS stage I	1（10%）
II	2（20%）
III	7（70%）
Number of plasma cells ≥30%	9（90%）
Complicated with nephropathy	2（20%）
Complicated with extramedullary lesions	1（10%）
Cytogenetics/FISH	
Standard risk	3（30%）
High risk: del(17p), t(4;14), t(14;16)	7（70%）

Table 2 The detail information of 6 known lncRNA and 6 Novel lncRNAs

Gene id	Gene name	Gene location	Log2FoldChange	*p-value*	Status	Regulation
ENSG00000225783	MIAT	chr22:26646428-26676475:+	1.583219127	0.0002	lincRNA	up
ENSG00000242094	FOXP1-IT1	chr3:71570255-71574457:-	-1.859445454	0.000210685	sense_intronic	down
ENSG00000248323	LUCAT1	chr5:91303029-91314402:-	-3.590904852	1.12E-05	lincRNA	down
ENSG00000226471	CTA-292E10.6	chr22:28800683-28848559:+	1.766039974	4.65E-05	antisense	up
ENSG00000245105	A2M-AS1	chr12:9065177-9068060:+	-2.5297359	7.56E-09	antisense	down
ENSG00000269821	KCNQ1OT1	chr11:2608328-2699994:-	1.482539004	0.00022904	antisense	up
MSTRG.260088	Novel	chr8:33555498-	2.164	1.16E-05	intronic	up
MSTRG.155519	Novel	chr20:20428784-20441936:-	-2.6722	4.13E-11	intronic	down
MSTRG.110981	Novel	chr17:58655474-58659291:-	2.08356	0.003425	intronic	up
MSTRG.190620	Novel	chr4:9594836-9604897:-	2.3591	0.013972752	lincRNA	up
MSTRG.193521	Novel	chr4:40956005-40964940:-	2.31858	0.009353052	intronic	up
MSTRG.13132	Novel	chr1:117632351-117682714:+	2.895250636	2.14E-07	lincRNA	up

Table 3. The detail information of 3 known miRNAs

Gene id	Log2FoldChange	p-value	Status	Regulation
hsa-miR-345-5p	-2.287909855	4.85E-06	Known miRNA	down
hsa-miR-193b-3p	3.598865128	1.11E-06	Known miRNA	up
hsa-miR-338-5p	-2.915272871	0.003110253	Known miRNA	down
